# Development of Neuroendocrine Prostate Cancers by the Ser/Arg Repetitive Matrix 4-Mediated RNA Splicing Network

**DOI:** 10.3389/fonc.2018.00093

**Published:** 2018-04-03

**Authors:** Ahn R. Lee, Nicole Che, Jessica M. Lovnicki, Xuesen Dong

**Affiliations:** Vancouver Prostate Centre, Department of Urologic Sciences, University of British Columbia, Vancouver, BC, Canada

**Keywords:** alternative RNA splicing, androgen receptor pathway inhibition, castration-resistant prostate cancer, lineage plasticity, neuroendocrine prostate cancer, resistant mechanisms, Ser/Arg repetitive matrix 4

## Abstract

While the use of next-generation androgen receptor pathway inhibition (ARPI) therapy has significantly increased the survival of patients with metastatic prostate adenocarcinoma (AdPC), several groups have reported a treatment-resistant mechanism, whereby cancer cells can become androgen receptor (AR) indifferent and gain a neuroendocrine (NE)-like phenotype. This subtype of castration-resistant prostate cancer has been termed “treatment-induced castration-resistant neuroendocrine prostate cancer” (CRPC-NE). Recent reports indicate that the overall genomic landscapes of castration-resistant tumors with AdPC phenotypes and CRPC-NE are not significantly altered. However, CRPC-NE tumors have been found to contain a NE-specific pattern throughout their epigenome and splicing transcriptome, which are significantly modified. The molecular mechanisms by which CRPC-NE develops remain unclear, but several factors have been implicated in the progression of the disease. Recently, Ser/Arg repetitive matrix 4 (SRRM4), a neuronal-specific RNA splicing factor that is upregulated in CRPC-NE tumors, has been shown to establish a CRPC-NE-unique splicing transcriptome, to induce a NE-like morphology in AdPC cells, and, most importantly, to transform AdPC cells into CRPC-NE xenografts under ARPI. Moreover, the SRRM4-targeted splicing genes are highly enriched in various neuronal processes, suggesting their roles in facilitating a CRPC-NE program. This article will address the importance of SRRM4-mediated alternative RNA splicing in reprogramming translated proteins to facilitate NE differentiation, survival, and proliferation of cells to establish CRPC-NE tumors. In addition, we will discuss the potential roles of SRRM4 in conjunction with other known pathways and factors important for CRPC-NE development, such as the AR pathway, *TP53* and *RB1* genes, the FOXA family of proteins, and environmental factors. This study aims to explore the multifaceted functions of SRRM4 and SRRM4-mediated splicing in driving a CRPC-NE program as a coping mechanism for therapy resistance, as well as define future SRRM4-targeted therapeutic approaches for treating CRPC-NE or mitigating its development.

## Introduction

Prostate cancer (PCa) is not just a singular disease; it is many diseases that are interconnected through molecular, phenotypic, and functional heterogeneity not only between patients but also within the individual. This heterogeneity is one of the greatest challenges in developing therapeutic programs for PCa. Heterogeneity arises during the development of the cancer through genetic, epigenetic, post-transcriptional, and post-translational alteration events in the tumor. In various malignancies, the fusion of both genetic and epigenetic adaptations promotes the cell to undergo processes of cellular plasticity, such as dedifferentiation or transdifferentiation, which in turn increases the rate of tumor growth, promotes resistance to therapeutics, and facilitates invasion and metastasis (1–5).

Clonal evolution theories suggest that random mutations and clonal selection generate the cellular heterogeneity seen in cancers (6, 7). This model is supported by the genetic diversity of subclones seen in primary and metastatic tumors of various cancers, including PCa (4). However, the mechanism by which this diversity in malignant cells emerges to form different subtypes of cancer remains unknown. Several mechanisms of heterogeneity establishment have been proposed including the capability of cancer cells to exhibit a remarkable degree of plasticity and the existence of cancer stem cells (1–5), although it is still controversial whether true cancer stem cells exists in PCa (8). In this section, we highlight the most recognized and supported mechanisms of lineage plasticity to promote tumor growth, metastasis, invasion, survival, and treatment resistance in PCa, with specific emphasis on the neuroendocrine (NE) prostate cancer (NEPC) subtype.

### Heterogeneity of Castration-Resistant Prostate Cancer (CRPC)

The primary treatment for locally advanced or metastatic PCa is androgen receptor pathway inhibition (ARPI). This treatment is normally effective for many patients, but the benefits are short-lived as the cancer inevitably progresses to a more lethal CRPC status (9–12). Although more potent, new generation ARPI therapies, such as enzalutamide and abiraterone acetate, have been shown improved patient survival, resistance to these therapies inevitably occurs (13, 14). Overall, there are three main classifications of resistance mechanisms to ARPI that have been demonstrated to date: androgen-dependent AR signaling, receptor-dependent AR signaling, and bypass of AR signaling. In androgen-dependent AR signaling, tumor cells can restore the AR signaling pathway by increasing the synthesis of circulating androgens (15, 16, 17) or by acquiring AR gene overexpression, amplification, and mutations that allow AR activation by attenuated levels of androgens following castration or ARPI (18–22). By contrast, tumor cells can re-gain active AR signaling that is independent of androgen ligand-mediated activation of the AR by means of generating constitutively active splice variants of the AR (23–26), altering the mode of actions of the AR in a receptor-dependent manner (27), or by relying on the downstream signaling of other hormone receptor pathways, such as the glucocorticoid receptor (28). CRPC tumors that restore their AR signaling retain their luminal epithelial or adenocarcinoma (AdPC) phenotypes and are referred to as castration-resistant adenocarcinoma prostate cancer (CRPC-Ad) tumors. However, a subset of tumor cells will develop mechanisms that help them to bypass their dependency on the AR signaling altogether and progress into AR “indifferent” tumors. One subtype of AR indifferent CRPC that exhibits NE phenotypes is called treatment-induced castration-resistant neuroendocrine prostate cancer (CRPC-NE) (29–31). Generally, NEPC is defined by the expression of NE markers, such as synaptophysin (*SYP)*, chromogranin A (*CHGA)*, and neuronal-specific enolase (*NSE)*, and the loss or low expression levels of epithelial makers, such as E-cadherin (*CHD1*), PSA (*KLK3*), and *AR* (32). Another subtype of AR indifferent CRPC that was recently reported by Bluemn et al. (33) is a double-negative AR-null and NE marker-null CRPC. Moreover, FGF and MAPK pathways have been reported to drive tumor progression, whereby activating the FGF pathway can bypass AR signaling and promote ARPI resistance in tumor cells.

Castration-resistant prostate cancer tumors do not exclusively use one of the three different mechanisms of ARPI resistance. It has been well established that CRPC tumors exhibit a varied range of AR expression levels, resulting in a significant degree of phenotypic, functional, and molecular heterogeneity seen within a tumor (8, 32). Furthermore, histopathological heterogeneity in the expression levels of various markers and genes has also been reported. For example, in AdPC tumors, NE foci (that are positive for NE markers) are observed in anywhere from 10 to 100% of the tumors examined (34–37). However, most of the tumors with NE differentiation were not confirmed to progress to CRPC-NE. In fact, it was recently demonstrated that a new genetically engineered CRPC mouse model with co-inactivation of *TP53* and *PTEN*, named *NPp53*, progresses to either CRPC-Ad tumors with NE foci that are non-proliferative or CRPC tumors explicit with NE differentiation that are highly proliferative, suggesting the importance of active proliferative genes during CRPC progression for the CRPC-NE phenotype to emerge (38).

Based on these observations in PCa, we hope that one can appreciate the vast degree of histopathological, molecular, phenotypic, and functional heterogeneity seen within not only CRPC subtypes but also individual NEPC cases. In fact, heterogeneity extends to the different subtypes of NEPC, such as AdPC with NE differentiation, AdPC with Paneth cell NE differentiation, carcinoid tumors, and small or large cell NEPC, which are classified by their histopathological characteristics (39). Furthermore, within individual CRPC-NE tumors, the expression levels of AR, as well as the expression of different NE markers, vary (40). This observation suggests that there are many complex mechanisms involved in the development of CRPC-NE; however, due to the limited understanding of the molecular underpinnings of NEPC development, the NEPC markers of detection and its various subtypes have not been well defined in the clinic or in the lab. Unfortunately, there is no gold standard to diagnose NEPC. Currently, in the clinic, SYP, CHGA, and NSE are the three main NE cells markers used to histologically detect NEPC tumors; although it has been shown that 10–40% of AdPC tumors are positive for these same markers, which demonstrates the relatively poor specificity of these diagnostic markers (41).

Overall, these findings confirm that CRPC heterogeneity develops as a result of survival mechanisms as a means to escape treatments, such as ARPI, either by progressing into AR-driven AdPC, AR “indifferent” CRPC-NE, or double-negative AR-null and NE marker-null PCa tumors.

## Mechanisms of Lineage Plasticity

Lineage plasticity of PCa cells represents one of the greatest challenges in PCa therapeutics. It is described as a mechanism of ARPI resistance, whereby PCa cells with luminal epithelial phenotypes gain the ability to transform into other lineages or phenotypes, such as NE cell lineages. To date, several articles have reported that lineage plasticity can be used as a survival mechanism for cells to develop ARPI resistance by progressing into either AR-driven CRPC-Ad or AR “indifferent” CRPC-NE (32, 40, 42–44). How PCa cells gain this lineage plasticity and choose which lineage fate remains unclear; however, recent studies have demonstrated that genetic, epigenetic, and RNA splicing regulations, as well as tumor microenvironment factors, may influence the plasticity of PCa cells.

### Genetic and Epigenetic Modifications Confer Plasticity of PCa Cells

Although the overall global genomic landscape (i.e., somatic copy number, point mutations, and polyploidy) between CRPC-Ad and CRPC-NE shows a significant overlap, some genetic alterations contribute to the lineage plasticity of PCa cells to produce the heterogeneity seen within PCa (40, 45). For example, loss-of-function alterations in *TP53, RB1*, and *PTEN* tumor suppressor genes are a common and frequent occurrence in CRPC-NE compared with changes in CRPC-Ad (46). Recent studies have also shown that a double knockdown of both *RB1* and *TP53* genes in the human LNCaP AdPC cell line facilitates resistance to ARPI (44). Furthermore, these cells display a degree of plasticity with increased expressions of basal epithelial and NE cell markers, as well as a decrease in expression of luminal epithelial cell markers (44). This article proposed a model of lineage plasticity in luminal epithelial cells, whereby cells undergo reprogramming and dedifferentiation from a luminal to a NE-like basal or mesenchymal lineage as a result of SOX2 deregulation, which is a putative developmental factor essential for self-renewal and pluripotency (44). Moreover, recent articles have reported the role of BRN2, a POU-domain transcription factor known to promote neuronal differentiation during neurogenesis, in driving NE differentiation of ARPI-resistant AdPC cells *via* SOX2 regulation (44, 47). This study demonstrated that BRN2 could promote augmented NE marker expression to drive a neural program together with SOX2 in ARPI-resistant AdPC cells to promote CRPC-NE development. In addition, a recent study by Zou et al. (38) revealed that the genetically engineered *PTEN-* and *TP53-*loss mouse model called NPp53 recapitulated human CRPC progression, whereby the tumors progressed quicker to this phenotype following ARPI treatment rather than show a positive response to the treatment. Following their analyses, these tumors had highly aggressive and proliferative phenotypes and displayed histopathological phenotypes similar to treatment-induced CRPC-NE. This group suggested that SOX11, a known target of p53 and found to be conserved in CRPC-NE tumors, could be responsible for promoting neuronal differentiation downstream of SOX2, which may be required earlier on to promote epithelial plasticity during CRPC-NE progression. In fact, predicted targets of SOX11 are BRN2 and N-Myc, which have also been shown to be drivers of CRPC-NE. This suggests that an initial establishment of epithelial plasticity and a degree of potency by early factors such as SOX2 and subsequent, downstream factors such as BRN2 and N-Myc are important in coordinating the NE cell lineage fate to promote the formation of CRPC-NE. These key findings suggest a role of the tumor suppressor genes *TP53, RB1*, and *PTEN*, as well as the essential temporal regulation of the SOX family of transcription factors, such as SOX2 and SOX11, in driving lineage plasticity of PCa cells.

Although there is large overlap in genomic landscapes between CRPC-Ad and CRPC-NE, there are significant differences in the epigenomic profiles of these two types of cancers. It is suggested that this marked difference in the genome-wide DNA methylation status between CRPC-Ad and CRPC-NE tumors is primarily driven by a histone methyltransferase called EZH2, where both its protein and mRNA levels are upregulated in CRPC-NE tumors (40, 48, 49). Recent findings by Dardenne et al. (50) have shown that N-Myc and EZH2 signaling activity is tightly coupled to drive a CRPC-NE molecular program (50). This study reported that the overexpression of N-Myc, a neuronal-specific transcription factor highly enriched in ~40% of CRPC-NE tumors and is associated with a range of neural cancers, increases EZH2 activity, which in turn, represses AR signaling and promotes an enhanced activation of the PI3K/AKT pathway to drive CRPC-NE development. Although further studies are required, these results suggest a potential mechanism by which N-Myc can promote an EZH2-mediated reprogramming of the epigenome to drive CRPC-NE development. In addition, Ku et al. (43) demonstrated that the lineage transition and ARPI resistance seen in the *RB1* and *TP53* double knockdown cell line is induced by EZH2, and treatment with EZH2 inhibitors could reverse this phenomenon (43). To date, these findings have shown the significance and interplay of genetic alterations and epigenetic modifications in driving the lineage plasticity, and thus, the emergence of CRPC-NE.

### Alternative RNA Splicing Confers Plasticity of PCa Cells

Alternative mRNA splicing events in cancer cells have been found to facilitate the aggressive behaviors of cancers, which have been previously reviewed (51). In the context of PCa, it has been shown that tumor cells exploit splicing processes to promote tumor plasticity, treatment resistance, tumor growth, proliferation, and differentiation (44). For example, AR splicing products, such as ARv7 (the most frequent variant of AR observed), have been shown to promote the resistance to ARPI and the proliferation of various cell line models in a ligand-independent, constitutively active manner (52–56). Various RNA splicing regulators have also been shown to change expression patterns in PCa. One of these regulators, Sam68, has been shown to be upregulated in PCa and promote cell survival and metastasis (57). For the purpose of this article, we will focus on a splicing regulator and driver of cellular plasticity in PCa cells and CRPC-NE development called Ser/Arg repetitive matrix 4 (SRRM4).

Lapuk et al. (58) have previously shown that DNA and RNA sequencing of CRPC-Ad and CRPC-NE tumors have an increased expression of SRRM4, a neural-specific mRNA splicing factor, which was unique to CRPC-NE (58). A follow-up study performed with deeper whole-transcriptome analyses on two CRPC-Ad/NE patient cohorts identified a CRPC-NE-specific RNA splicing signature that is predominately driven by SRRM4 (59). In their article, SRRM4 is responsible for the aberrant splicing of at least 16 key target genes involved in the transformation of AdPC to CRPC-NE under ARPI (59). Furthermore, SRRM4 is shown to drive the transformation of LNCaP AdPC to CRPC-NE xenografts when inoculated under the renal capsule of castrated mice. In addition, long-term studies of serial passaging subcutaneously inoculated LNCaP cells overexpressing SRRM4 in castrated mice generated a series of five treatment-induced CRPC-NE xenograft models called LnNE (60). As these tumors were passaged, they showed an increased expression of NE markers, grew more aggressively, and exhibited a decreased or no PSA expression, thus, mimicking characteristics of AdPC progression to treatment-induced CRPC-NE. Furthermore, recent findings have elucidated the role of SRRM4 in inducing NE-like phenotypes in an array of various cell types, such as PCa stromal cells, benign prostate hyperplasia cells, and normal prostate cells (Lee et al., unpublished manuscript). In this study, all of the cell lines overexpressing SRRM4 show an overall increase in NE markers and a decrease in AdPC markers, although heterogeneity in the expression of some markers were seen within the different cell line models overexpressing SRRM4. In addition, all cell lines express the CRPC-NE-specific RNA splicing signature previously reported in CRPC-NE tumors (59). These findings strongly support that SRRM4 drives NE differentiation of PCa cells and CRPC-NE tumorigenesis in a cell context-dependent manner.

## Ser/Arg (SR) Family Proteins: SRRM4

Ser/Arg repetitive matrix 4 belongs to a family of proteins involved in mRNA splicing called serine/arginine (SR)-related proteins. These proteins have a diverse range of functions in facilitating alternative splicing of genes, which, in turn, can have dramatic effects on the function, localization, stability, and/or expression of differentially spliced mRNA or its resulting translated protein (61). To fully elucidate the vast roles of SRRM4 in the pathological development of CRPC-NE, we must first identify and understand the SR family and SR-related family of proteins, as well as the normal biological and molecular functions of SRRM4 during neuronal development.

### The SR Family and SR-Related Family of Proteins

It has been determined that 90–98% of the genes in the human body have alternative splice variants, emphasizing the importance that alternative splicing plays a critical role in normal development (62, 63). In fact, it has been proposed that alternative RNA splicing is the source of biological diversity and complexity within the human neural system (64, 65). The interactions between *cis*- and *trans*-acting factors are essential in regulating alternative splicing through either the repression or activation of splice-site selection. The factors essential for orchestrating splicing programs include RNA-binding domain-containing small nuclear ribonucleoproteins (also known as snRNP), such as U1, U2, U4, U5, and U6 [reviewed by Kramer (66), Will and Luhrmann (67)], and SR family and SR-related family of proteins (66, 67). These components make up a macromolecular complex called the spliceosome. The SR family of proteins contains one or two conserved RNA recognition motifs (RRM) at the N-terminus that are essential for RNA-binding specificity, as well as an arginine/serine-rich (RS) domain of varying sizes at the C-terminal end, which is important for promoting the protein–protein interactions and recruitment of the spliceosome complex (68, 69). The SR family of proteins has diverse functions in regulating not only pre-mRNA splicing but also post-splicing events, such as the exportation of mRNA, nonsense-mediated mRNA decay, and the translation of mRNA, which has been previously reviewed (70, 71). On the other hand, SR-related proteins contain an RS domain, but may or may not contain a defined RRM (72). Similar to SR family of proteins, SR-related proteins are found to play a role in not only splicing, but in other fundamental cellular processes, such as chromatin remodeling, cell cycle progression, and transcription (73). The regulation of SR protein splicing activity and its subcellular localizations, in part, depend on the dynamic cycle of phosphorylation and dephosphorylation of the serine residues in its RS domain (74). The phosphorylation statuses of RS proteins have a diverse effect in mediating the regulation of spliceosome complex assembly, recruitment, splicing activation, and splice-site selection *via* alterations to the protein–protein and protein–RNA interactions (75–77).

While SR proteins have many similar characteristics, SRRM4 (also known as nSR100) is particularly interesting as it has been suggested to be the source of proteomic diversity and functional complexity of the vertebrate nervous system, although its evolutionary origin is unclear (78). SRRM4 was identified as a neural tissue- and vertebrate-restricted SR protein involved in complex alternative splicing of neural-specific exons, which are essential for vertebrate nervous system development and neural cell fate differentiation (78). In addition, SRRM4 is uncharacteristically heavy, weighing 100 kDa, which is likely a result of a large RS region in the protein. The presence of large RS domains makes SRRM4 more highly phosphorylated than its family members, an important characteristic of splice-site selection. Furthermore, the RS-rich domains of SRRM4 have also been predicted to be responsible for protein–protein and/or protein–RNA interactions required for spliceosomal complex assembly to promote splicing that allows SRRM4 to regulate brain-specific exon inclusion of genes associated with neuronal development (68, 79–81). In conclusion, SR proteins play an essential role in regulating development and key cellular processes, suggesting that the dysfunction of these proteins will result in unsuccessful development and abnormal cellular processes, which can lead to human diseases and cancers.

### SRRM4 in Normal Development

Raj et al. (80) recently demonstrated that by knocking down or overexpressing SRRM4 *in vivo*, SRRM4 mediates the inclusion of 30–50% of conserved mouse and human brain-specific exons, manifesting their unique neural-splicing profiles (80). *In utero* SRRM4 knockdown studies in mice demonstrated a diminished differentiation of neural progenitors (82). Furthermore, knockdown of SRRM4 in neural cells and in zebrafish demonstrated impaired neurite morphogenesis and branching (78). In Calarco et al.’s (78) work, it was also revealed that SRRM4-mediated inclusion of neural-specific exons of genes encoding neuronal GTPase activity, which have putative roles in cytoskeletal remodeling and dendritic growth and branching, can alter protein-coding sequences, suggesting that these target exons may stimulate protein–protein interaction networks. Another study showed that an inactivating mutation in the *SRRM4* gene caused deafness and balance impairments in Bronx Waltzer mice (83). Importantly, transcriptomic analysis of the sensory neurons in these mice exhibited a distorted splicing signature when compared to normal mice (83). These findings suggest an essential role of SRRM4 function during neural development to promote neural cell fate differentiation, in particularly, its function in the splicing of neural-specific exons to regulate the overall resulting protein function from its processed pre-mRNA.

A specific, widely studied mechanism that heavily relies on SRRM4 function during neurogenesis to support that SRRM4 is implicated in the splicing of neural-specific exons is that it facilitates neuronal differentiation *via* a cross-regulatory mechanism with a transcriptional repressor called REST (78, 80, 82, 84). REST is a transcriptional repressor that binds to the RE-1 site in the regulatory region, upstream of a target gene, and inhibits the transcriptional activity of neuronal genes by facilitating repressive histone modifications *via* the recruitment of co-repressors and factors, such as HDAC1/2, coREST, and Sin3A, to the gene’s promoter region. During neurogenesis, REST and SRRM4 antagonistically regulate the developmental process. Specifically, REST represses the transcription of genes that are important for driving the neuronal phenotype and has also been shown to directly inhibit SRRM4 expression as a means to prevent neurogenesis (82). Conversely, SRRM4 functions to facilitate the alternative splicing of *REST* into a splice variant called REST4. REST4 is a truncated isoform of the REST protein with reduced DNA-binding function. It has also been shown that REST4 isoforms can directly bind to REST to inhibit its function (84), thus, resulting in the transcriptional activity of REST-repressed target genes. In summary, high expression of REST suppresses the expression of neuronal-specific genes and inhibits SRRM4 activity in non-neuronal cells. On the other hand, to achieve neuronal cell differentiation, increased splicing activity of SRRM4 is required to increase the expression of the dominant-negative isoform REST4, which inhibits REST’s function to activate the transcription of genes important for neuronal development. The critical roles of these genes in fostering and regulating normal neural development has also been demonstrated in studies that investigate the consequences of mutations within or dysregulations of these genes, which will be described within this article. In conclusion, these findings strongly support a key role of SRRM4 as a critical regulator of neuronal development and normal neuronal cell function.

### SRRM4 in Pathogenesis

Based on the fundamental role of SRRM4 and its functions during normal neural development, it is clear that aberrant SRRM4 expression or function will promote pathogenesis. Aberrant RNA splicing has been demonstrated as a mechanism exploited by cells to promote the progression of many diseases, such as neurological diseases and cancers (44, 62, 81, 83, 85–87). In fact, SRRM4 has been associated with various diseases where the altered expression and splicing function of SRRM4 promotes the progression of these diseases, such as small cell lung cancer (SCLC), a NE cancer of the lung (59, 83, 88, 89). These neurological-related diseases with aberrant SRRM4 expression and function exhibited marked variations in their splicing programs. Therefore, studying the altered splicing profiles of diseases by SRRM4 will provide a new avenue to investigate the molecular mechanisms and outcomes of splicing during disease progression.

As mentioned earlier, our recent studies demonstrate that CRPC-NE tumors have a very conserved and unique splicing signature, where SRRM4 promotes the inclusion of microexons of genes that are highly enriched in neuronal functions (59). These results are supported by a previous study done on SRRM4 function and alternative splicing in autistic spectrum disorder (ASD) (85). In their study, Irimia et al. (85) observed a reduced expression of SRRM4 in ASD, which resulted in the dysregulation of the highly conserved SRRM4-mediated splicing program. Collectively, both studies revealed that the positions of the microexons in the SRRM4-targeted splice variants were near or overlapped regions of conserved domains and motifs that are important for protein–protein interactions during neural development (59, 85). This suggests that microexons are important in remodeling protein complexes and interaction networks, resulting in altered pathways to promote the neuronal lineage cell fate. To summarize, the consequences of the downstream spliced genes of SRRM4 were found to be important for several hallmarks in instigating CRPC-NE tumor establishment, such as driving NE lineage differentiation, stimulating neurite growth, evading apoptosis, promoting proliferation, and potentially regulating the epigenome. Further studies are essential in fully understanding the molecular mechanisms involved in CRPC-NE progression *via* SRRM4-mediated alternative splicing events.

## Roles of SRRM4 in CRPC-NE Development

Recently, it has been shown that CRPC-NE patient tumors develop a unique splicing profile when compared with the profiles of CRPC-Ad, whereby CRPC-NE tumors share 16 distinctly spliced genes that are primarily driven by SRRM4 (59). These spliced genes encode kinases that can activate major signaling cascades/pathways (such as the MAPK pathway), GTPases that can promote NE-specific morphogenesis, facilitators of proliferation and invasion, post-translational modifiers (such as acetylation and methylation), cell survival regulators, and neural differentiation fate factors. These CRPC-NE-specific spliced genes have recognized functions that are important for facilitating various aspects of neural programming in early development (80, 81). SRRM4-mediated RNA splicing of these genes includes, but is not limited to, *REST, Bif-1*, MYST/Esa1-associated factor 6 (*MEAF6*), and PHD finger protein 21A (*PHF21A*), all of which will be elaborated on how they promote CRPC-NE development downstream of SRRM4 in the following sections. However, it is also important to note that SRRM4 may facilitate CRPC-NE progression in conjunction with other known pathways and factors important for CRPC-NE development, such as the AR pathway, *TP53* and *RB1* genes, the FOXA family of proteins, and environmental factors. In this section, we will discuss how SRRM4 may be connected to multiple pathways of different known drivers of CRPC-NE progression and how SRRM4-mediated spliced variants facilitate the development of CRPC-NE tumors downstream of SRRM4.

### SRRM4, the Androgen Receptor (AR), p53, and RB1

While primary *de novo* NEPC makes up <1% of PCa incidences, the majority of PCa cases are AR-driven PCa. However, after therapeutic treatments such as ARPI, tumors will inevitably gain resistance to these AR-targeted therapies. As previously described, some tumors will restore their AR signaling and some, such as CRPC-NE, will become AR “indifferent.” The fact that CRPC-NE arises mainly after therapeutics shows that AR pathway regulation plays a fundamental role in the progression to CRPC-NE. In fact, sequencing studies revealed that nearly all CRPC-NE incidences arise as a result of the selection pressures of therapeutics (40, 44). Patient-derived xenograft CRPC-NE models reveal that AdPC tumors under ARPI conditions can transform to NEPC tumors (44, 45). Furthermore, studies have demonstrated that the AR pathway can suppress PCa lineage plasticity into NE or other cell lineages, where ARPI can reduce AR-mediated repression of SOX2 and induce BRN2, which in turn positively regulates the expression of SOX2, a fundamental regulator of stemness during embryonic development (47, 90, 91). These findings suggest a clear role of AR in conferring AdPC lineage plasticity and that the selection pressure of ARPI is imperative for the development of CRPC-NE.

Our lab has recently observed that SRRM4 mRNA is present in small populations of AdPC tumors, where the prevalence and mRNA expression of SRRM4 increases after therapeutic interventions such as ARPI in CRPC-Ad tumors, suggesting that active AR signaling can repress SRRM4 expression, and consequently function (Li et al., manuscript under review). In this study, we propose that one of the earliest initial molecular events in the emergence of SRRM4-driven CRPC-NE occurs in two possible ways. One way is that the existing SRRM4-positive population of PCa cells is selected for survival under the selection pressures of ARPI. Alternatively, prolonged ARPI can increase SRRM4 expression *via* epigenetic alterations regulated, in part, by AR. Although the specific molecular cross-talk mechanisms between AR and SRRM4 remain to be discovered, we hypothesize that, based on previous and current findings, the conjunction of ARPI and SRRM4 in the clinic promotes CRPC-Ad progression to CRPC-NE. However, it is noteworthy to add that PCa patients are treated with multi-therapeutics, where ARPI is commonly combined with chemo- or radiation therapies. Due to this, we cannot rule out other therapeutics that may contribute to the emergence of CRPC-NE.

The role of AR in conferring AdPC lineage plasticity has been shown to be augmented by functional inactivation of p53 and RB1 to promote the emergence of CRPC-NE. Recent whole-genome sequencing of CRPC-Ad and CRPC-NE tumors has revealed that ~55–75% of cases have concurrent functional mutations or deletions of the *RB1* and *TP53* genes, as opposed to the ~15–40% of cases seen in CRPC-Ad tumors (40, 44). This suggests that these genomic aberrations are highly correlated with CRPC-NE tumors and may play a role in the development of CRPC-NE. In fact, one of the earliest PCa transgenic mouse models, called TRAMP, demonstrated the implications of these tumor suppressors in the emergence of CRPC-NE tumors (92). This TRAMP mouse model expresses the transforming region of SV40 large T antigen, which acts to sequester and inactivate both p53 and RB1. These TRAMP mice spontaneously develop PCa that closely resembles the molecular and phenotypic characteristics and progression of hormone-naïve PCa to metastatic CRPC-Ad to CRPC-NE. However, the progression to CRPC-NE observed in the TRAMP mice models relied upon ARPI conditions. Indeed, recent research has demonstrated that ARPI treatment in conjunction with the loss of function of p53 and RB1 facilitates lineage plasticity of AdPC cells to basal, mesenchymal, or NE-like cells in various mice models (43, 93). A study by Mu et al. (94) demonstrated that the loss of function of p53 and RB1 in AdPC cells under ARPI conditions induced lineage plasticity, favoring the NE cell lineage *via* increased expression of SOX2 (44). However, these findings show that the acquired lineage plasticity by p53 and RB1 functional inactivation under ARPI is not limited to CRPC-NE specifically, suggesting other lineage directions. A prime example is brain metastasis-derived DU 145 AdPC cells that have inactivated mutations in *TP53* and *RB1* as well as an AR-null genomic profile. Furthermore, it is important to note the well-established function of p53 and RB1 in the emergence of CRPC-NE as a putative tumor suppressor of proliferation and survival. As their genomic and functional alterations are prevalent in CRPC-NE tumors, these alterations are important to the uncontrolled hyperproliferation observed in clinical NEPC tumors (95).

These findings suggest that, together with ARPI, inhibiting p53 and RB1 function increases lineage plasticity of PCa cells allowing for the differentiation of other cell types. In our recently generated CRPC-NE LnNE xenograft model, SRRM4-mediated transformation and tumor progression of AdPC tumors into CRPC-NE tumors under ARPI was augmented with the addition of *TP53* knockdown (59). Throughout serial passaging of the LnNE xenografts in castrated hosts, these tumors recapitulated the progression of CRPC-NE as the expression levels of AR and PSA decreased over time. Moreover, in our recent work, SRRM4-overexpressing DU 145 cell lines transform into a tumor that closely reproduces clinical CRPC-NE in histopathology and exhibits an increased nucleus to cytoplasm ratio, decreased cell size, and dendritic outgrowths in culture (Lee et al., unpublished manuscript). Interestingly, this striking morphological change from a luminal epithelial lineage to a NE cell lineage was only observed in DU 145 cells, which have a unique genomic profile of *AR*-null and *TP53* and *RB1* functional mutations. However, as mentioned earlier, p53 and RB1 functional mutations are characteristic to DU 145 cells where no NE lineage differentiation is observed, and any known drivers of CRPC-NE are not expressed, supporting that p53 and RB1 function in NE lineage cell fate is cell-context dependent. As the AR-mediated repression of SRRM4 function is irrelevant in DU 145 cells, we suggest that the aberrant functions of p53 and RB1 in DU 145 cells primes the cells to be more susceptible to SRRM4-driven NE lineage fate determination. We hypothesize that SRRM4 may induce the expression of key stemness regulator genes such as *SOX2* in DU 145 to promote lineage plasticity and NE lineage differentiation to drive CRPC-NE progression. Furthermore, SRRM4 may promote lineage plasticity *via* a different mechanism. SRRM4-mediated splicing of the *MEAF6* gene creates a CRPC-NE-unique MEAF6-1 splice variant that can increase the expression of the inhibitor of differentiation-1, or *ID1*, in PCa cells (96). It has been previously demonstrated that RB1 function can be indirectly inhibited by ID1 through inhibition of ETS-mediated p16 activation, which the signaling cascade results in the activation phosphorylation of RB1 (97, 98). These findings suggest that MEAF6-1-induced expression of the *ID1* gene may indirectly inhibit RB1 function to further promote the development of CRPC-NE tumors *via* their putative functions in uncontrolled cell proliferation. This will be further described in the latter sections. Overall, these findings suggest that SRRM4 plays a role in the lineage plasticity of AdPC cells to confer NE cell fate through a potential RB1-SOX2 pathway and splicing of the *MEAF6* gene, which may in turn inhibit RB1 function *via* ID1. Although the details in the underlying molecular mechanisms must still be elucidated, these findings suggest that the AR signaling pathway as well as *TP53* and *RB1* genomic aberrations are implicated in facilitating lineage plasticity in non-neuronal cells where the conjunction of SRRM4 function can drive the NE cell lineage fate to promote the emergence of CRPC-NE.

### SRRM4 and the FOXA Family of Proteins

The FOXA families of proteins are a family of transcription factors that have recently been implicated in the development of CRPC-NE. FOXA proteins are pioneer factors that modify and open chromatin to orchestrate the recruitment of transcription factors, such as the AR, to their intended target sites to regulate gene expressions (99). Two members of the FOXA family, such as FOXA1 and FOXA2, are important regulators of differentiation and development of the prostate during embryogenesis (100). Although very similar in structure, it was initially shown by Mirosevich et al. (100) that the functions carried out by these two proteins are unalike, as their temporal and spatial patterns are dissimilar during development (100). In this study, they report that FOXA2 is only expressed in the early stages of prostate development and only in a subset of cell within the basal layers, and colocalizes to cells positive for NE markers, whereas FOXA1 is expressed robustly throughout the development, growth, and adult differentiation of the prostate. Remarkably, the spatial and temporal patterns of FOXA1/2 expression were also observed in the TRAMP mouse model (101). This study detected FOXA1 expression in the normal prostate and throughout the progression of PCa, whereas FOXA2 was only expressed in the normal prostate. However, in the CRPC-NE progressed tumors of the TRAMP mouse model, FOXA2 was seen to be re-expressed (101). Supporting the findings of this study, a recent study done by Park et al. (102) revealed a unique expression of FOXA2 in PCa tumor microarrays of primary small cell NEPC as well as treatment-related CRPC-NE tumors (102). These findings are also consistent with previous findings of a positive correlation of FOXA2 expressions in NE cancers of the lung (103). Another recent study by Kim et al. (104) demonstrated that the inhibition of FOXA1 promoted NE differentiation in both PCa cell lines and mouse models (104). In this study, they also show that FOXA1 expression is downregulated in CRPC-NE tumor models such as the recently generated LnNE CRPC-NE xenograft model (58, 59). As mentioned earlier, this xenograft model was derived from LNCaP cells overexpressing SRRM4, suggesting a potential mechanism whereby SRRM4 may inhibit FOXA1 expression. Although further investigation is needed to fully elucidate the mechanism of FOXA1/2 in the NE differentiation of PCa cells, these findings suggest a contrasting role for FOXA1 and FOXA2 in regulating lineage plasticity where FOXA1 is involved in inhibiting NE-differentiation and promoting the differentiated state of AdPC cells. Furthermore, SRRM4 may regulate the expression of FOXA1 by inhibition to promote the emergence of CRPC-NE.

### SRRM4-Mediated Alternative RNA Splicing of Master Neural Differentiation Regulator *REST*

REST can achieve NE differentiation suppression in AdPC cells. REST, as mentioned earlier, is a suppressor of neurogenesis where it acts as a transcriptional repressor of neuronal genes, such as SYP, through the recruitment of corepressors and histone methylation modifiers. During neurogenesis, SRRM4 creates a splice variant called REST4, which has been shown to antagonize REST protein and reprogram REST functions, resulting in the removal of neuronal transcriptional suppression in non-neuronal cells (105). This developmental mechanism is exploited in AdPC cells under ARPI to promote the NE lineage cell fate (58, 59). In these studies, decreased expression of REST and increased expression of the splice variant REST4 were identified as a CRPC-NE-unique transcriptomic signature. To support these findings, a study done by Zhang et al. (89) completed a microarray analysis on CRPC patient tumor samples and patient-derived xenografts and showed a positive correlation with increased SRRM4 expression, loss of REST, and increased REST splicing and CRPC tumors with NE phenotypes (89). Similar to the mechanisms of neural developmental, SRRM4 was shown to promote NE differentiation of AdPC cells *via* direct binding and splicing of the *REST* gene and, in turn, inhibit the transcriptional repression of REST on neuronal genes *via* the dominant-negative function of the REST4 splice variant (59). These observations were also seen in SCLC, an NE cancer of the lung (88). Shimojo et al. (88) found that SRRM4 and SRRM4-target REST4 splice variant expression was high and REST expression was low in SCLC. The mechanisms by which SRRM4 regulated the alternative splicing in SCLC was similar to that in normal development (88). Apart from the SRRM4-mediated mechanism of RNA splicing to reprogram the function of REST to promote the emergence of CRPC-NE development, a recent study performed by Chen et al. (106) revealed that REST degradation or protein instability induced by PI3K/AKT pathway inhibition can facilitate a NE phenotype in AdPC cells which was observed by an overall increase in NE markers (106). Furthermore, the function of REST has been recognized to be an important facilitator of hypoxia-induced CRPC-NE progression as it is a master regulator of hypoxia genes, which will be discussed in the later section. In conclusion, research to date supports the critical role of REST in the emergence of CRPC-NE, where loss of REST-mediated repression on neuronal genes is important in facilitating the NE phenotype. The mechanisms in which REST is regulated has been shown at both the RNA and protein level, where SRRM4-mediated RNA splicing is key to the functional inactivation of REST and inhibition of the PI3K/AKT pathway promotes the degradation of the REST protein. Based on these current findings, it is clear that REST plays a critical role to the development and differentiation of CRPC-NE.

### SRRM4-Mediated Alternative RNA Splicing of Apoptosis Regulator *Bif-1*

Neuroendocrine prostate cancer, like any other cancer, requires more than just differentiation to develop—resistance to cell death caused by ARPI, radiation, or chemotherapies is the prerequisite condition for CRPC-NE to develop. In fact, the Bax-interacting factor 1 (*Bif-1* or *SH3GLB1*) gene is spliced in CRPC-NE tumors (59). Bif-1 is an endophilin protein involved in apoptosis, autophagy, and mitochondrial functions. The function of Bif-1 is mediated *via* interactions with different co-factors and proteins such as UVRAG and Beclin-1, which form autophagosomes, regulate mitochondrial dynamics, and facilitate tumorigenesis (107). In addition, Bif-1 can interact with Bax to cause a conformational change to functional Bax activation *via* its N-BAR domain in response to apoptotic stress to promote mitochondrial membrane permeability for cytochrome *c* release (107, 108). The N-BAR domain of the Bif-1 protein is required for both the activation of Bax and promotion of mitochondrial lipid membrane remodeling (109). The *Bif-1* gene has three main splice variants, where the Bif-1a splice variant is expressed in all tissues and the Bif-1b and Bif-1c splice variants are brain tissue specific (110). In non-neuronal cells, Bif-1 promotes apoptosis under stress conditions; however, Bif-1 in neuronal cells promotes neuronal viability by increasing mitochondria membrane stability and inhibiting the release of cytochrome *c* (111). Interestingly, the inclusion of microexons 6 and 7 in the *Bif-1b* and *Bif-1c* genes, respectively, are within the N-BAR domain of the *Bif-1* gene (111). This suggests that the insertion of the microexons may potentially perturb protein–protein interactions or other related functions of the N-BAR domain of the Bif-1b and Bif-1c spliced variants, which may alter the function of Bif-1 into an anti-apoptotic function. Based on these findings and known functions of Bif-1 and its splice variants, we hypothesize that SRRM4 mediates the splicing of Bif-1 into CRPC-NE-unique splice variants Bif-1b and Bif-1c, and that these variants are important in helping PCa cells, particularly CRPC-NE cells, to escape apoptosis during CRPC-NE tumor progression. By contrast, we posit that the constitutive isoform, Bif-1a, which is highly represented in CRPC-Ad tumors, is a pro-apoptotic facilitator. Understanding the functions of Bif-1 splice variants will be important in elucidating the multifaceted molecular mechanisms in which SRRM4 and its RNA splicing activity drives the progression of CRPC-NE.

### SRRM4-Mediated Alternative RNA Splicing of Epigenomic Regulators *MEAF6* and *PHF21A*

In addition to the role of SRRM4 in evading apoptosis *via* alternative splicing of the *Bif-1* gene, it has recently been demonstrated that SRRM4 can promote the proliferation of cells *via* splicing of the *MEAF6* gene (96). Among the CRPC-NE-unique gene signatures found by Li et al. (59), *MEAF6* gene was differentially spliced, and the splice variant MEAF6-1 was uniquely manifested in CRPC-NE tumors (59). MEAF6 is a component of four of five MYST families of histone acetyltransferase (HAT) complexes. HAT complexes have putative roles in key fundamental nuclear processes (i.e., transcription, DNA repair, and replication) *via* post-translational modifications of histones and transcriptional regulators, such as p53. These essential functions and components of HAT complexes are evolutionarily conserved from yeast to humans (112–114). This suggests and has been previously demonstrated that the deregulation or altered functions of the components that comprise the HAT complexes play an important role in cancer progression by altering the cancer epigenome. In fact, it was recently demonstrated by Lee et al. (96) that the neural-specific variant of the *MEAF6* gene, MEAF6-1, but not non-neural splice variant MEAF6-2 promoted cell proliferation (96). This was confirmed by BrdU incorporation assay’s under 2D and 3D matrigel conditions. In addition, MEAF6-1 promoted colony formation, in colony number and size, and invasion and migration of PCa cells. Furthermore, MEAF6-1 promoted tumor growth in mice. Interestingly, microarray analyses showed that the functions of MEAF6-1 were mediated by ID1 and ID3, which was validated *in vivo* by silencing these genes in AdPC cells stably expressing MEAF6-1. Collectively, this study demonstrated that MEAF6-1, but not MEAF6-2, promotes cell proliferation, invasion, and migration of PCa cells as well as tumor growth of xenograft models. As MEAF6 is a component of HAT complexes, it would be interesting for future research to study any potential variations in the protein–protein interactions between MEAF6 splice variants and the components of the HAT complexes and the function of the MEAF6-1 splice variant at the epigenomic level.

Another regulator of histone modifications that is a target of SRRM4 unique to CRPC-NE tumors is the *PHF21A* gene (58). PHF21A is a component of the histone modification complexes associated with LSD1, coREST, and HDAC1/2, which suppress the transcription of neuronal genes. As the DNA-binding activity of the complexes relies on the concurrent functions of both PHF21A and LSD1, knockdown of PHF21A results in the de-repression of LSD1 target genes (115–117). It was also previously shown that PHF21A functions as a negative modulator of REST-mediated repression of neuronal genes *via* inhibition binding to the REST protein (117). The human *PHF21A* gene encodes a neural tissue-specific splice variant in which the exon 12a is included, which has been shown to be a CRPC-NE-unique splice variant, and a ubiquitously expressed or constitutive variant in which the exon 12, not exon 12a, is included (58, 118). Previously, Iwase et al. (119) demonstrated that the neural-specific PHF21A splice variant had an increased binding to HDAC1/2 when compared with the PHF21A constitutive isoform (119). In this study, it was proposed that RNA alternative splicing of the PHF21A gene can reprogram the function of the PHF21A protein by altering the protein–protein interactions of PHF21A, HDAC1/2, and potentially other proteins. It is noteworthy to add that the constitutive PHF21A variant may facilitate altered histone deacetylation or demethylase activity as the inclusion of the alternatively spliced exon also disrupts an AT hook responsible for DNA binding, as well as one of the two predicted nuclear localization signals in the *PHF21A* mRNA (120). This supports the idea that the inclusion of the alternatively spliced exon facilitated by SRRM4 can reprogram protein function by altering protein–protein interactions or potentially altering the localization of the protein (85). Furthermore, the resulting reprogrammed function of the SRRM4-mediated PHF21A splice variant may also attenuate the transcriptional repression of REST and its associated cofactors, LSD1, coREST, and HDAC1/2 on neuronal-specific genes, whereby lifting the REST-dependent histone methylation repression of neuronal differentiation and increasing the likelihood of NE lineage cell differentiation in PCa cells.

Based on these findings, it is clear that SRRM4 mediates the splicing of genes that are CRPC-NE-specific and known to be highly enriched during neurogenesis. These newly spliced genes are important for promoting CRPC-NE tumor establishment, where the reprogrammed functions of CRPC-NE-unique MEAF6-1 splice variants can drive PCa cell proliferation, invasion, tumor growth, and, along with the PHF21A constitutive splice variant, may drive a neural program at the epigenomic level. Although further studies are needed to investigate the role of these splice variants in epigenomic modifications to facilitate CRPC-NE development, the fact that CRPC-NE-specific genes targeted by SRRM4 splicing are important epigenetic modifiers suggests that SRRM4 may function at the epigenetic level.

### SRRM4 and the Microenvironment

Although further studies are required to elucidate the complexity of the mechanisms associated with environmental factor-mediated emergence of CRPC-NE, studies to date have suggested that the microenvironment is an important inducer of lineage plasticity and differentiation to the NE cell lineage. As previously discussed, stress factors from therapeutics such as ARPI have been demonstrated to induce CRPC-NE. Studies have revealed other various environmental factors important in NEPC development such as cAMP (121, 122), cytokines (i.e., IL6 and IL8) (123, 124), AKT inhibition (106), and hypoxia (125, 126). However, the induction of the NE phenotype by environmental factors such as cAMP (121, 122) is reversible, suggesting that other factors and molecular mechanisms are essential to the development of CRPC-NE tumors.

Alternative factors in the tumor stromal microenvironment that promote the emergence of CRPC-NE are mitogenic cytokines, such as IL8 and IL6 (104, 123, 124, 127). Early studies by Huang et al. (127) observed that benign NE cells in the prostate express high levels of IL8 (127). They also demonstrated that the expression of IL8 receptor, CXCR1, increased from low-to-none in benign epithelial cells to high in high-grade PCa to higher in metastatic PCa. This suggests a paracrine mechanism, whereby IL8 secretion from NE cells may stimulate the growth and proliferation of adjacent non-NE tumor cells. By contrast, a follow-up study demonstrated that NE cells express a different IL8 receptor called CXCR2, whereby NE cell quiescence is induced *via* the IL8–CXCR2–p53 pathway in an autocrine fashion in which mutant p53 can promote hyperproliferation of cells through inactivation of this signaling pathway (95). Parallel to this finding, a recent study by Kim et al. (104) reported that the inhibition of FOXA1, which is a transcriptional repressor of the *IL8* gene, promotes NE differentiation in both AdPC cell lines and mouse models by IL8-mediated activation of MAPK/ERK pathway and thus transcriptional activation of NE markers (104). Collectively, these findings demonstrate the implications of autocrine or paracrine IL8-mediated signaling pathways in promoting the emergence of NEPC. Furthermore, a mechanistic connection between mutant p53 and IL8, both of which are prevalent in CRPC-NE tumors, may drive hyperproliferation to promote the emergence of CRPC-NE tumors. Another important cytokine that has been shown to promote NE differentiation is IL6 (123). In fact, it has recently been demonstrated that IL6 can induce NE differentiation in LNCaP cells by suppressing REST function, whereas exogenous REST abolished the IL6-induced NE program (124). In this study, they also demonstrated that IL6-induced NE differentiation promoted REST protein degradation *via* the ubiquitin–proteasome pathway. Another study reported that REST function is essential for IL6-induced NE differentiation (128). Both IL6 and REST have both been implicated in hypoxia-induced NE differentiation (126). Hypoxia is another important regulator of NE differentiation in PCa cells. Interestingly, hypoxia-induced NE differentiation of PCa cells relies on the inhibition of REST, as REST is a master repressor of neuronal genes and thus a regulator of hypoxia-induced genes (126, 129). These studies suggest a possible mechanism of tumor microenvironment factors such as hypoxia, cytokines, and REST functions in promoting NE differentiation. Furthermore, research by Qi et al. (130) demonstrated that ubiquitin ligase Siah2-expressing TRAMP mice was required for hypoxia-mediated CRPC-NE development (130). In this study, it was revealed that Siah2 regulation of HIF1a activity, a master regulator of hypoxia, which has been shown to be highly expressed in CRPC-NE tumors, is essential for the transcriptional activation of HIF1a/FOXA2 target genes such as SOX9 to promote the NE phenotype of CRPC-NE tumors. These findings indicate possible broader mechanistic pathway of NE differentiation that may involve the FOXA2–HIF1a hypoxia pathway and IL6-induced REST inhibition in concurrence with SRRM4-induced REST splicing and inhibition *via* REST4 function under stress conditions. Connecting these pathways together, it is possible that SRRM4 may contribute to the FOXA2–Saih2/HIF-1a pathway, where inhibition of REST function can be augmented by both SRRM4 function and FOXA to drive the NE lineage cell fate in AdPC cells. Consequently, it is possible that SRRM4 may act in conjunction or synergy with tumor microenvironment factors or regulators to further drive the development and progression of CRPC-NE.

## SRRM4 as a Therapeutic Target for NEPC

Based on research findings, many factors are essential during CRPC-NE development. Research strongly suggest that ARPI treatment, genomic alterations, alterations in expression and function of histone modification enzymes, RNA splicing factors, and transcriptional factors are necessary for reprogramming the cell to gain lineage plasticity. The plethora of factors involved in CRPC-NE development emphasizes the heterogeneity of NEPC. Therefore, further investigation is needed to understand how these signaling pathways interplay with each other and understand the molecular mechanisms by which these factors promote CRPC-NE. Ultimately, this knowledge will provide insight for personalized medicine-based strategies for PCa patients. These efforts will rationalize SRRM4 and its CRPC-NE-unique splice variants as potential diagnostic or prognostic biomarkers, and SRRM4 as a therapeutic target in CRPC-NE patients.

In fact, SRRM4 fulfills several criteria to be a possible therapeutic target. Although knockdown of the *SRRM4* gene has been shown to be critical during neural development (78, 80, 82), a partial deletion of 710 bp in the C-terminus of the *SRRM4* gene did not result in embryonic lethality in Bronx Waltzer mice (83). In these mice, the deletion impaired the splicing activity of SRRM4, but resulted in limited abnormal neural behaviors, such as deafness and impaired balance. This suggests that specific inactivation of the SRRM4 splicing activity while maintaining the majority of the protein may have limited side effects. Furthermore, SRRM4 is prominently expressed in neuronal cells and CRPC-NE tumors, but is rarely expressed in non-neuronal cells. SRRM4-specific inhibitors for CRPC-NE must be carefully designed to prevent entering the blood–brain barrier to limit off-target effects of SRRM4.

There are several ways to block SRRM4 function such as antisense oligonucleotides (ASO) or splice-switching oligonucleotides (SSO), which can redirect SRRM4-mediated alternative splicing events. Alternatively, small molecule inhibitors (SMI) can either target the RNA-binding domain of SRRM4 to prevent it from recognizing its RNA substrates or can directly inhibit the C-terminus domains important for the splicing activity of SRRM4. In fact, the first Food and Drug Administration-approved ASO/SSO called SPINRAZA™ has shown to be effective in patients with spinal muscular atrophy, a genetic disorder whereby altered RNA splicing patterns of the survival motor neuron (SMN) genes results in an unstable and dysfunctional SMN protein (131, 132). SPINRAZA™ targets the splicing events of the SMN genes by silencing the splicing silencer element in the intron region upstream of the alternatively spliced exon. Although further mechanistic studies are warranted, a similar method may be applied to mitigating CRPC-NE progression, whereby carefully designed ASO/SSO could inhibit SRRM4-recognized consensus RNA-binding UGC motifs in Ref. (80). Furthermore, although the exact mechanism of splicing inhibition is currently unknown, SMI compound called LMI070 is currently under clinical trials to treat spinal muscular atrophy, by binding to the RNA itself or RNA-binding domains of splicing factors (131). Before SRRM4-targeting SMI drug design, the SRRM4 protein must be crystalized *via* microfluidics reaction technology or cryo-electron microscopy methods. However, these techniques face many challenges, such as large amount of protein purification and low resolution, respectively. Following SRRM4 crystallization, SMI that bind to either the RNA-binding domain or the C-terminus domain may be designed. These methods highlight the potential for SRRM4 to be used as a target for future CRPC-NE therapeutic programs.

## Conclusion

**Figure 1:**
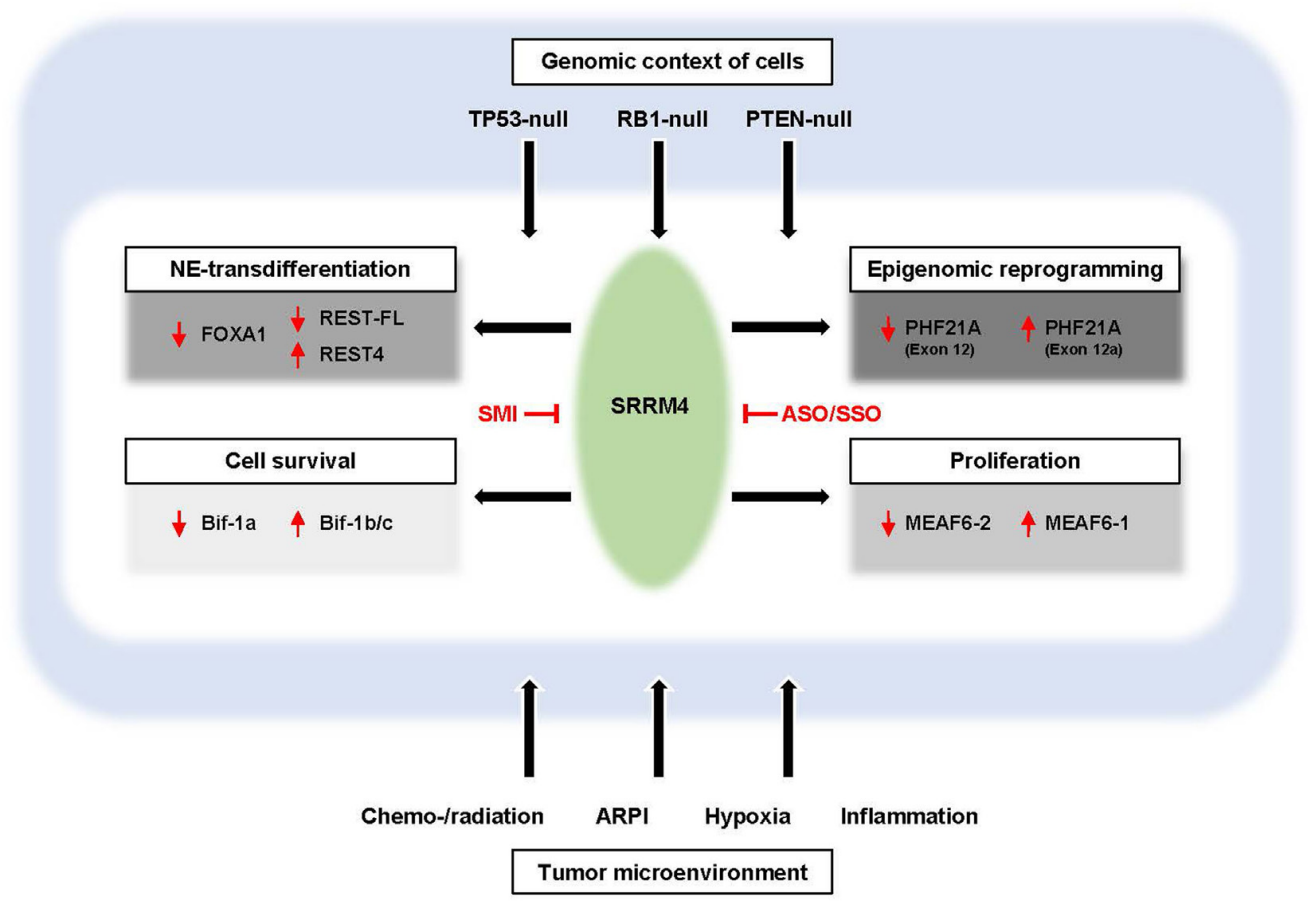
Development of NEPC by the SRRM4-mediated RNA splicing network. The multifaceted roles of SRRM4 and SRRM4-mediated alternative RNA splicing of genes highly enriched in neuronal functions drives a CRPC-NE program *via* various aspects important for CRPC-NE progression. SRRM4-mediated spliced variants not only facilitate the NE-transdifferentiation of CRPC-Ad cells possibly through epigenetic alterations but also promotes cancer cell survival, proliferation, and tumorigenesis of CRPC-NE cells to establish CRPC-NE tumors. We propose that SRRM4 may function beyond its putative role in RNA splicing *via* mechanisms involving transcriptional factors (e.g., FOXA, REST, and AR), tumor suppressors (e.g., RB1 and p53), and microenvironment factors (e.g., therapeutics, hypoxia). Potential SRRM4-targeted therapeutic approaches for treating CRPC-NE or mitigating its development may be to inhibit the splicing or RNA-binding activity of SRRM4 *via* SMI or target the alternative splicing events *via* ASO. This multi-functional property of SRRM4 ultimately provides cancer cells the ability to develop therapy resistance and develop into CRPC-NE tumors. SMI, small molecule inhibitors; ASO, antisense oligonucleotides; SSO, splice-switching oligonucleotides; ARPI, androgen receptor pathway inhibition; CRPC-Ad, castration-resistant adenocarcinoma prostate cancer; NEPC, neuroendocrine prostate cancer; SRRM4, Ser/Arg repetitive matrix 4.

SRRM4 is an important driver gene for CRPC-NE. By regulating alternative RNA splicing, SRRM4 not only stimulates AdPC to undergo NE differentiation but also promotes cancer cell survival, proliferation, and tumorigenesis, illustrated in Figure 1. SRRM4 also interacts with other signal pathways including AR, p53, and RB1 to regulate phenotypical reprogramming PCa cells. This multi-functional property of SRRM4 ultimately provides cancer cells the ability to develop therapy resistance and develop into CRPC-NE. The information gathered for this article on SRRM4 in driving CRPC-NE progression will strengthen the rationale to design SRRM4 inhibitors that are expected to be effective to treat SRRM4-driven CRPC-NE.

## Author Contributions

AL contributed to the conception and design of the article and performed the necessary and required literature research to the drafting of the work. NC contributed to the conception by writing the first draft of the work. All authors revised the work critically for important intellectual content and contributed to manuscript revision, read, and approved the submitted version.

## Conflict of Interest Statement

The authors declare that the research was conducted in the absence of any commercial or financial relationships that could be construed as a potential conflict of interest.

## Abbreviations

PCa, prostate cancer; NE, neuroendocrine; NEPC, neuroendocrine prostate cancer; AR, androgen receptor; ARPI, androgen receptor pathway inhibition; CRPC, castration-resistant prostate cancer; AdPC, prostate adenocarcinoma; CRPC-Ad, castration-resistant adenocarcinoma prostate cancer; CRPC-NE, treatment-induced castration-resistant neuroendocrine prostate cancer; SR, serine/arginine; RRM, RNA recognition motifs; RS, arginine/serine-rich; SCLC, small cell lung cancer; ASD, autistic spectrum disorder; HAT, histone acetyltransferase; SMI, small molecule inhibitors; ASO, antisense oligonucleotides; SSO, spice-switching oligonucleotides.
